# Assessment of MRI susceptibility-weighted imaging-based liver-to-muscle signal intensity ratios for the staging of liver fibrosis

**DOI:** 10.1186/s13244-025-02203-2

**Published:** 2026-01-28

**Authors:** Xuan Jin, Yufan Ren, Xuchang Zhang, Haojun Lu, Jiaqi Lv, Tianyuan Zhang, Wen Liang, Yongzhou Xu, Qing Yu, Xianyue Quan, Xinming Li

**Affiliations:** 1 Department of Radiology, The Fourth Affiliated Hospital of School of Medicine, and International School of Medicine, International Institutes of Medicine, Zhejiang University, Yiwu, China; 2 Department of Radiology, Zhujiang Hospital, Southern Medical University, Guangzhou, China; 3 Department of Medical Imaging Center, Nanfang Hospital, Southern Medical University, Guangzhou, China; 4 Medical Imaging Center, The First People’s Hospital of Foshan, Foshan, China; 5 Philips Healthcare, Guangzhou, China; 6 Department of Radiology, Heyou Hospital, Foshan, China

**Keywords:** Susceptibility-weighted imaging, Liver fibrosis, Serological biomarkers, Magnetic resonance imaging

## Abstract

**Objective:**

To investigate the feasibility of susceptibility-weighted imaging (SWI) for the diagnosis of different stages of liver fibrosis, and to assess its diagnostic accuracy compared with the serum fibrosis index commonly used in clinical settings.

**Materials and methods:**

This prospective study included 108 patients and 16 healthy volunteers. All patients underwent MRI with SWI and histopathological evaluation. Liver and bilateral erector spinae signal intensities were measured on SWI to calculate liver-to-muscle signal intensity ratios (SIR). Serological biomarkers were collected to calculate the aspartate aminotransferase-to-platelet ratio index (APRI) and fibrosis index based on four factors (FIB-4). Histological correlation analysis between the SIR and liver fibrosis/iron deposition was performed using Spearman’s rank correlation analysis. The diagnostic accuracies of SIR, APRI, and FIB-4 for staging liver fibrosis were assessed, and their performances were compared using the DeLong test.

**Results:**

Receiver operating characteristic (ROC) curve analysis showed good-to-excellent diagnostic performance of SIR for different stages of liver fibrosis. The areas under the curve (AUC) of SIR for the diagnosis of liver fibrosis stages S0 vs S1–S4, S0–S1 vs S2–S4, S0–S2 vs S3–S4, and S0–S3 vs S4 were 0.851, 0.868, 0.872, and 0.931. Delong’s test showed that the SIR outperformed the APRI and FIB-4 in the diagnosis of liver fibrosis S0–S1 vs S2–S4, S0–S2 vs S3–S4, and S0–S3 vs S4 (*p* = 0.011–0.036).

**Conclusion:**

SWI-based SIR outperforms the serum indicators APRI and FIB-4 in diagnosing liver fibrosis of S0–S1 vs S2–S4, S0–S2 vs S3–S4, and S0–S3 vs S4.

**Critical relevance statement:**

SWI-based SIR offers a new perspective on non-invasive diagnostic methods to guide the clinical diagnosis of liver fibrosis, particularly in cases where biopsy is contraindicated or impractical.

**Key Points:**

- Searching for a non-invasive method to accurately diagnose stages of liver fibrosis is necessary because of the limitations of histopathological evaluation.
- SWI offers a dependable and non-invasive diagnostic approach for evaluating different stages of liver fibrosis compared to serological biomarkers.
- SWI-based SIR provides a highly accurate, non-invasive alternative to serum biomarkers for detecting advanced liver fibrosis.

**Graphical Abstract:**

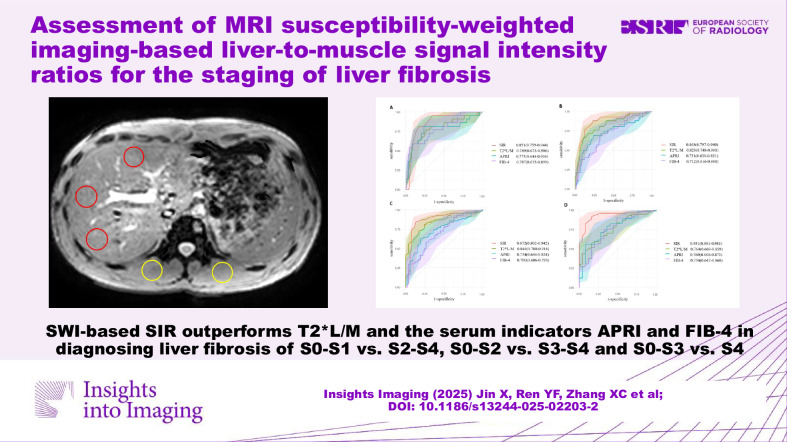

## Introduction

Liver fibrosis is a highly prevalent chronic liver condition globally [1]. Oxidative stress associated with this condition can impair hepatic metabolic function, potentially leading to abnormal hepatic iron deposits [2]. Iron deposition partially contributes to the advancement of liver fibrosis, which is a critical step toward cirrhosis and could eventually result in hepatocellular carcinoma [3]. The progression of liver fibrosis negatively impacts the patient’s quality of life and is associated with poor prognoses [4]. Notably, previous research has shown that early-stage liver fibrosis can be reversed [5]. Consequently, early detection of the disease’s extent is pivotal for guiding treatment decisions [6]. Although histopathological evaluation is the definitive method for diagnosing and staging liver fibrosis, it has notable limitations, including its invasive nature and the risk of multiple complications [7, 8]. These challenges have prompted growing interest in using magnetic resonance imaging and serum fibrosis indices for staging liver fibrosis.

Shear wave elastography and Magnetic resonance elastography are recommended for dynamically measuring liver stiffness [9]. However, Shear wave elastography can be confounded by both pathologic and normal physiologic processes [10], and Magnetic resonance elastography requires specialized equipment and is unsuitable for patients with significant iron overload [11]. Although serum fibrosis indices can be readily obtained, their clinical utility is limited [12] due to factors such as etiology and the presence of inflammation or infection [13]. Additionally, the use of artificial intelligence to evaluate the progression of liver fibrosis shows promise. However, challenges remain regarding the development of these complex models, and the generalizability of these tools [14]. Therefore, exploring simple and reliable non-invasive assessment approaches is essential for the accurate diagnosis of early liver fibrosis.

Susceptibility-weighted imaging (SWI) can enhance image contrast by detecting subtle differences in tissue magnetization [15, 16]. Additionally, SWI offers advantages such as shorter breath-hold times and faster acquisition times. Previous studies have shown that SWI possesses high sensitivity and specificity in detecting hepatic iron deposition [17], which is closely associated with liver fibrosis progression [18, 19]. Despite advances in the application of SWI for detecting liver iron deposition [20], its potential for staging liver fibrosis requires further study. Researchers have demonstrated that SWI effectively detects and stages liver fibrosis in rabbits [21] and rats [22]. However, relatively few studies have examined the correlation between SWI, iron deposition, and liver fibrosis in humans.

The aim of this study was to investigate the feasibility of SWI for the diagnosis of different stages of liver fibrosis and to compare its diagnostic performance with the serum fibrosis index commonly used in clinical practice, providing a new perspective for easy non-invasive evaluation of liver fibrosis staging.

## Material and methods

### Study population

**Fig. 1:**
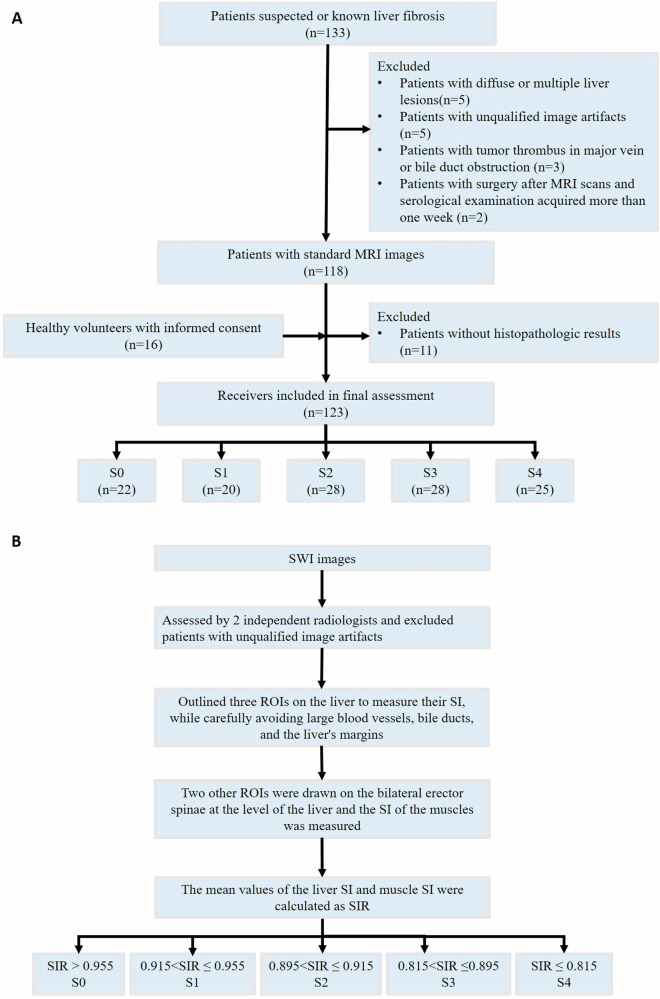
**A** Flowchart of overall cases. **B** Flowchart of clinical workflow using the SWI-based SIR for fibrosis staging. ROI, region of interest; SI, signal intensity; SIR, liver-to-muscle signal intensity ratios; S0–S4, liver fibrosis stages according to the Scheuer scoring system; SWI, susceptibility-weighted imaging

This single-center prospective study received approval from the local ethics committee (Zhujiang Hospital, Southern Medical University, Guangdong Province, China, reference number 2017-YXZDK-002), and all participants provided informed consent. Between January 2018 and December 2021, 133 patients were initially identified. The inclusion criteria for patients with liver fibrosis were: (a) patients suspected or known to have liver fibrosis; (b) no prior surgery or local treatment before the MRI scans and serological examination; (c) age ≥ 18 years. The exclusion criteria were as follows: (a) diffuse or multiple liver lesions, including hemochromatosis and fatty liver disease; (b) unqualified image artifacts, (c) tumor thrombus in a major vein or bile duct obstruction, (d) histopathological evaluation after MRI scans and serological examination acquired for more than one week, and (e) no histopathological evaluation. The study also included 16 healthy volunteers as a control group who were evaluated by clinicians and confirmed to have no liver fibrosis. The inclusion criteria were as follows: (a) no contraindications for MRI scans, (b) no recent abnormalities on serological examination, and (c) no diffuse liver disease or obvious large liver lesions. The exclusion criteria were as follows: (a) unqualified image artifacts. Finally, we included 107 consecutive patients with liver fibrosis and 16 healthy volunteers. Figure 1 presents a flowchart detailing inclusion and exclusion criteria. This prospective study was registered at ClinicalTrials. gov (NCT03176797).

### MR imaging

Both patients and volunteers underwent examination with a single imaging machine, a 3.0-T MRI scanner (Ingenia, Philips) equipped with an abdominal phased-array coil after fasting for 4 h before the procedure. The SWI settings were as follows: repetition time (TR) = 100 ms, echo time (TE) = 10 ms, flip angle = 20°, field of view (FOV) = 40 × 35 cm, matrix size = 160 × 140, voxel size = 2.5 × 2.5 × 5 mm3, slice thickness = 5 mm, number of signals averaged (NSA) = 1. The acquisition time for each sequence was 15 s during a single breath hold. Blood oxygen level-dependent (BOLD) MRI was used for hepatic imaging to quantify T2* relaxation times. The parameters were as follows: pulse sequence: multi-echo gradient echo, TR = 46 ms, TE = 2.3/4.6/6.9/9.2/11.5/13.8/16.1 ms, flip angle = 15°, FOV = 40 × 35 cm, and slice thickness = 5 mm. Routine sequences with T2-weighted imaging (T2WI), were also acquired for anatomical evaluation with the following parameters: T2WI (respiratory-triggered): TR = 514 ms, TE = 70 ms, FOV = 32 cm × 38 cm, matrix size = 180 × 160, slice thickness = 6 mm, NSA = 1.

### Image analysis

**Fig. 2:**
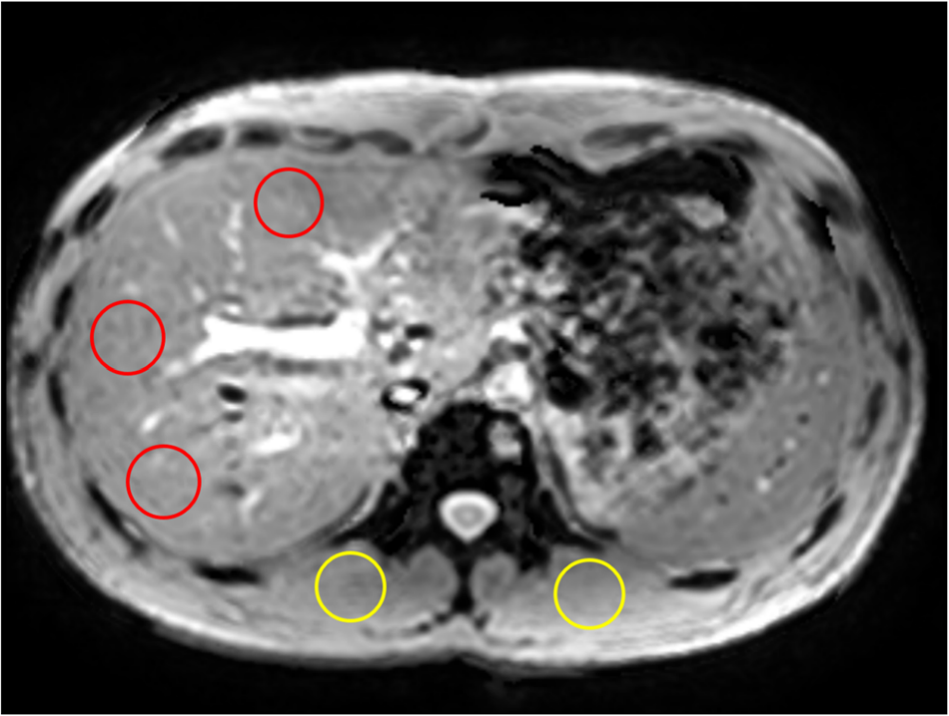
A diagram of SWI postprocedure processing. This was a 26-year-old male participant with a S0 liver fibrosis stage according to histopathological evaluations. The ROIs circled in red of the liver, avoiding large vessels, bile ducts, and the border, as well as the ROIs circled in yellow on both sides of the lumbar back muscles, were shown in unenhanced axial SWI

All SWI images were assessed by independent radiologists with 5 years (X.J.) and 3 years (Y.R.) of experience in liver MRI imaging. Images with poor quality or obvious artifacts were excluded. Both radiologists were blinded to the patients’ histopathological findings. They outlined three regions of interest (ROI) on the liver to measure their signal intensity (SI), while carefully avoiding large blood vessels, bile ducts, and the liver’s margins. Two other ROIs were drawn on the bilateral erector spinae at the level of the liver, and the SI of the muscles was measured. The area of all ROIs was maximized on the same image section and was no less than 3 cm^2^ (Fig. 2). We calculated the mean values of the liver SI and muscle SI as liver-to-muscle signal intensity ratios (SIR). The formula was as follows: SIR = average liver SI/muscle SI. Consistent with the abovementioned methodology, ROIs were delineated on both the liver and bilateral erector spinae to measure T2* values. The mean T2* values for the liver and muscle were subsequently computed, followed by the liver-to-muscle T2* ratio (T2* L/M) calculation using the following formula: T2* L/M = average liver T2*/muscle T2*.

### Serological biomarker collection

Clinical information and serum indices, including age, sex, platelet count (PLT), total bilirubin levels, aspartate aminotransferase (AST), alanine aminotransferase (ALT), international normalized ratio, and serum creatinine, were collected within one week prior to the MRI scan. The fibrosis index based on four factors (FIB-4) and aspartate aminotransferase-to-platelet ratio index (APRI) were calculated to assess liver function using the following formulas: APRI = [AST(U/L)/ULN] × 100/PLT(10^9^/L); FIB-4 = [age(years) × AST(U/L)]/[PLT(10^9^/L) × (ALT(U/L)) ^1/2^].

### Histopathological evaluations

Tissue slices of pathological liver specimens were stained with Masson’s trichrome and Perls’ Prussian blue. Liver fibrosis was staged using the Scheuer scoring system [23]. This system classifies the stages as follows: S0, absence of fibrosis; S1, fibrous portal expansion; S2, periportal or rare portal-portal septa; S3, fibrous septa with architectural distortion; and S4, cirrhosis.

A scoring system for liver iron content [24] was used to evaluate the grade of liver iron deposition, which includes five degrees as follows: Grade 0, none, absence of granules at high-power field (×400 magnification); Grade 1, minimal, granules clearly visible at ×400 magnification or faintly visible at ×250 magnification; Grade 2, mild, granules clearly visible at ×100 magnification; Grade 3, moderate, distinct granules seen at ×25 magnification; and Grade 4, severe, masses seen at low power (×10 magnification) or even without magnification. In cases of heterogeneous iron distribution, the highest observed grade was used.

### Statistical analysis

Categorical variables are represented as counts or percentages, whereas continuous variables are expressed as either means with standard deviations or medians with interquartile range. The variation between the evaluations of the two radiologists was assessed using the intraclass correlation coefficient (ICC), with values exceeding 0.75 indicating strong reliability. Inter-observer agreement for ROI measurements was evaluated using Bland–Altman analysis. Spearman’s correlation analysis was conducted to evaluate the association between the SIR and liver fibrosis stages. Distribution and variation in SIR across different stages of liver fibrosis were visualized through box-and-whisker plots. One-way analysis of variance was employed to determine statistically significant differences in SIR across different stages of liver fibrosis; an identical analytical approach was applied to T2* L/M. The diagnostic performance of the SIR, T2* L/M, APRI, and FIB-4 for staging liver fibrosis was assessed using receiver operating characteristic (ROC) curve analysis. The DeLong test was applied to compare the diagnostic performance of different approaches for assessing liver fibrosis. DeLong’s test *p*-values were adjusted using the Holm–Bonferroni method to control the family-wise error rate associated with multiple comparisons. All subsequent results of DeLong’s test and their interpretations are based on these values. All statistical tests were performed using SPSS software (version 27, IBM SPSS, Chicago, IL, USA), R software (version 4.4.1), and Origin software (version 2021, OriginLabxz). The *p*-value for statistical significance was < 0.05.

## Results

### Participants characteristics

A total of 123 participants were recruited for this study. According to the Scheuer scoring system, 22, 20, 28, 28, and 25 participants were diagnosed in groups S0–S4. All participants included in this study were free of comorbidities, such as non-alcoholic fatty liver disease (NAFLD) and hepatitis C virus. Table 1 provides a summary of participant characteristics for each liver fibrosis stage. The final cohort had a median age of 52 years, with an age range from 21 to 81 years, comprising 93 males and 30 females. The cohort comprised 91 patients with hepatocellular carcinoma or other malignancies, 16 with benign hepatic lesions, and 16 healthy volunteers.

**Table 1:** Characteristics of participants in our study

Parameters	Result
Gender (%)	
Male	93 (75.6%)
Female	30 (24.4%)
Age (years)	52 (42, 63)
HBsAg/HBV DNA status	
Positive	75 (61.0%)
Negative	48 (39.0%)
Cause of disease (%)	
Benign lesions^a^	16 (13.0%)
Malignant lesions^b^	91 (74.0%)
No disease	16 (13.0%)
AST (U/L)	25.00 (18.00, 38.35)
ALT (U/L)	26.00 (16.00, 37.00)
PLT (10^9^/L)	200.00 (155.00, 262.00)
TBil (μmol/L)	12.20 (8.40, 14.80)
INR	1.04 (0.99, 1.09)
Liver fibrosis staging (%)	
S0	22 (17.9%)
S1	20 (16.3%)
S2	28 (22.8%)
S3	28 (22.8%)
S4	25 (20.3%)
Necroinflammantory activity (%)	
G0	16 (13.0%)
G1	22 (17.9%)
G2	40 (32.5%)
G3	38 (30.9%)
G4	7 (5.7%)

*HBsAg* hepatitis B surface antigen, *HBV* hepatitis B virus, *AST* aspartate aminotransferase, *ALT* alanine aminotransferase, *PLT* blood platelet, *TBil* total bilirubin, *INR* international normalized ratio

^a^ Benign lesions include focal nodular hyperplasia (*n* = 6), angiomyolipoma (*n* = 1), hemangiomas (*n* = 1), lesions associated with hepatitis or cirrhosis (*n* = 6), lymphoepithelial lesions (*n* = 1), and hepatic fibroproliferative nodules (*n* = 1)

^b^ Malignant lesions include those associated with hepatocellular carcinoma (*n* = 83), cholangiocarcinoma (*n* = 5), combined hepatocellular-cholangiocarcinoma (*n* = 1), and metastatic tumor (*n* = 2)

### Observer agreement analysis

**Fig. 3:**
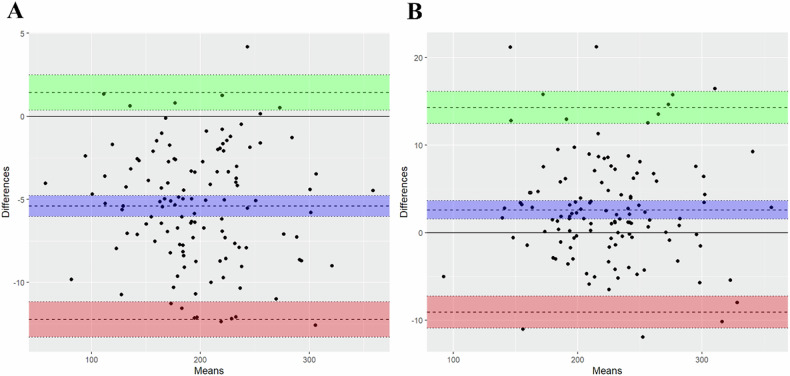
Bland–Altman plots for two radiologists. **A** Distribution of liver-SI measured by different observers. **B** Distribution of muscle-SI measured by different observers. SI, signal intensity. The agreement of measurements between the two observers was excellent, with relatively more variation in liver measurements than in bilateral erector spinae measurements

ICC analysis showed excellent agreement between the ROIs of the two radiologists outlining the liver and bilateral erector spinae on SWI: 0.993 (95% CI: 0.762–0.998) for liver measurement and 0.990 (95% CI: 0.982–0.994) for bilateral erector spinae measurement. Bland–Altman plots (Fig. 3) showed agreement in measurements between the two observers, with relatively greater variation in liver measurements than in bilateral erector spinae measurements.

### Differentiation analysis and one-way analysis of variance of SIR and T2* L/M

Box-and-whisker plot of SIR and T2* L/M across liver fibrosis stages demonstrated that SIR and T2* L/M decreased with increasing fibrosis staging (Fig. S1). The median SIR values for stages S0, S1, S2, S3, and S4 were 0.985, 0.945, 0.920, 0.855, and 0.770, and the median T2* L/M values for stages S0, S1, S2, S3, and S4 were 1.155, 1.080, 0.950, 0.770, and 0.690, respectively (Table S1).

One-way analysis of variance results for SIR and T2* L/M indicated that no significant difference was found between stages S0 and S1 (*p* = 0.944), or between stages S2 and S3 (*p* = 0.214) of SIR. Meanwhile, no significant difference was observed between stages S0 and S1 (*p* = 0.730), or between stages S3 and S4 (*p* = 0.521) of T2* L/M (Table S2).

### Diagnostic values for liver fibrosis staging

**Fig. 4:**
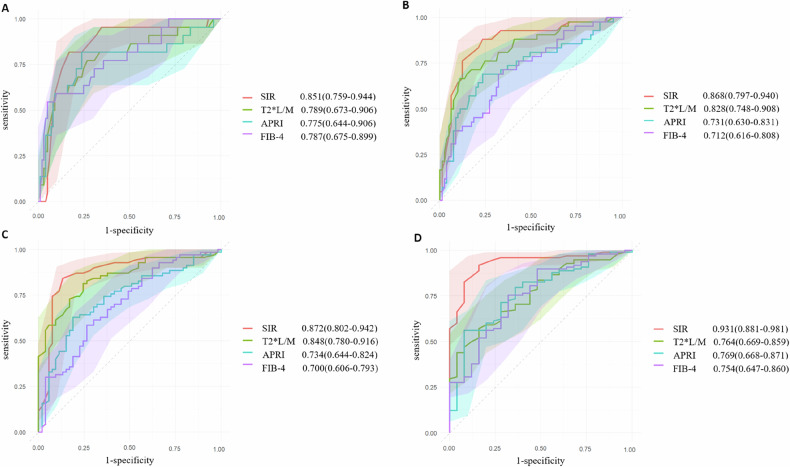
The ROC of SIR, T2* L/M, and serological biomarkers in different stages of liver fibrosis. ROC curves for SIR, T2* L/M, and serological biomarker values determined on the basis of two fibrosis stage thresholds: **A** S0 vs S1–S4. **B** S0–S1 vs S2–S4. **C** S0–S2 vs S3–S4. **D** S0–S3 vs S4. ROC, receiver operating characteristic curve; SIR, liver-to-muscle signal intensity ratios; S0–S4, liver fibrosis stages according to the Scheuer scoring system; T2* L/M, liver-to-muscle ratios of T2*; APRI, aspartate aminotransferase to platelet ratio index; FIB-4, fibrosis index based on four factors. The ROCs depict that the diagnostic efficiency of SIR is better than T2* L/M as well as serological biomarkers in most stages of liver fibrosis

ROC curve analysis showed good-to-excellent diagnostic performance of SIR for different stages of liver fibrosis (Fig. 4). The area under curves (AUC) of the SIR for the diagnosis of liver fibrosis stages S0 vs S1–S4, S0–S1 vs S2–S4, S0–S2 vs S3–S4, S0–S3 vs S4 were 0.851 (95% CI: 0.759–0.944), 0.868 (95% CI: 0.797–0.940), 0.872 (95% CI: 0.802–0.942), and 0.931 (95% CI: 0.881–0.981), respectively, and the optimal cut-off values were determined based on the Youden index as 0.955, 0.915, 0.895, and 0.815, respectively. Furthermore, the AUCs of T2* L/M, APRI, and FIB-4 for diagnosing the different stages of liver fibrosis were 0.789–0.848, 0.731–0.775, and 0.700–0.787, respectively. Tables 2 and 3 present the diagnostic values of the SIR, T2* L/M, APRI, and FIB-4 index for each liver fibrosis stage.

**Table 2:** Diagnostic values of SIR and T2* L/M for each stage of liver fibrosis

Parameters	SIR				T2* L/M			
	S0 vs S1–S4	S0–S1 vs S2–S4	S0–S2 vs S3–S4	S0–S3 vs S4	S0 vs S1–S4	S0–S1 vs S2–S4	S0–S2 vs S3–S4	S0–3 vs S4
AUC (95% CI)	0.851 (0.759–0.944)	0.868 (0.797–0.940)	0.872 (0.802–0.942)	0.931 (0.881–0.981)	0.789 (0.673–0.906)	0.828 (0.748–0.908)	0.848 (0.780–0.916)	0.764 (0.669–0.859)
ACC (95% CI)	0.829 (0.827–0.832)	0.805 (0.802–0.807)	0.854 (0.852–0.856)	0.902 (0.901–0.904)	0.740 (0.737–0.743)	0.813 (0.811–0.815)	0.789 (0.786–0.791)	0.626 (0.622–0.630)
SEN (95% CI)	0.818 (0.657–0.979)	0.881 (0.783–0.979)	0.843 (0.758–0.928)	0.918 (0.864–0.973)	0.773 (0.598–0.948)	0.667 (0.524–0.809)	0.814 (0.723–0.905)	0.571 (0.473–0.669)
SPE (95% CI)	0.832 (0.759–0.905)	0.765 (0.673–0.858)	0.868 (0.777–0.959)	0.840 (0.696–0.984)	0.733 (0.646–0.819)	0.889 (0.820–0.957)	0.755 (0.639–0.871)	0.840 (0.696–0.984)
PPV (95% CI)	0.514 (0.349–0.680)	0.661 (0.537–0.785)	0.894 (0.820–0.968)	0.957 (0.917–0.998)	0.386 (0.242–0.530)	0.757 (0.619–0.895)	0.814 (0.723–0.905)	0.933 (0.870–0.996)
NPV (95% CI)	0.955 (0.911–0.998)	0.925 (0.862–0.988)	0.807 (0.705–0.909)	0.724 (0.561–0.887)	0.937 (0.883–0.990)	0.837 (0.759–0.915)	0.755 (0.639–0.871)	0.333 (0.217–0.450)
Cut-off value	0.955	0.915	0.895	0.815	0.995	1.040	0.875	0.925

*SIR* liver-to-muscle signal intensity ratios, T2* L/M the liver-to-muscle ratios of T2* parametric values, *S0–S4* liver fibrosis stages according to the Scheuer scoring system, *AUC* area under curve, *ACC* accuracy, *SEN* sensitivity, *SPE* specificity, *PPV* positive predictive value, *NPV* negative predictive value

**Table 3:** Diagnostic values of APRI and FIB-4 for each stage of liver fibrosis

Parameters	FIB-4				APRI			
	S0 vs S1-S4	S0–S1 vs S2–S4	S0–S2 vs S3–S4	S0–S3 vs S4	S0 vs S1–S4	S0–S1 vs S2–S4	S0–S2 vs S3–S4	S0–3 vs S4
AUC (95% CI)	0.787 (0.675–0.899)	0.712 (0.616–0.808)	0.700 (0.606–0.793)	0.754 (0.647–0.860)	0.775 (0.644–0.906)	0.731 (0.630–0.831)	0.734 (0.644–0.824)	0.769 (0.668–0.871)
ACC (95% CI)	0.878 (0.876–0.880)	0.683 (0.679–0.686)	0.650 (0.647–0.654)	0.740 (0.737–0.743)	0.772 (0.770–0.775)	0.740 (0.737–0.743)	0.707 (0.704–0.711)	0.634 (0.630–0.638)
SEN (95% CI)	0.950 (0.908–0.993)	0.679 (0.577–0.781)	0.736 (0.617–0.855)	0.680 (0.497–0.863)	0.762 (0.679–0.845)	0.765 (0.673–0.858)	0.811 (0.706–0.917)	0.920 (0.814–1.000)
SPE (95% CI)	0.545 (0.337–0.754)	0.690 (0.551–0.830)	0.586 (0.470–0.701)	0.755 (0.670–0.840)	0.818 (0.657–0.979)	0.690 (0.551–0.830)	0.629 (0.515–0.742)	0.561 (0.463–0.659)
PPV (95% CI)	0.906 (0.850–0.961)	0.809 (0.715–0.902)	0.574 (0.456–0.691)	0.415 (0.264–0.565)	0.951 (0.903–0.998)	0.827 (0.741–0.912)	0.623 (0.509–0.738)	0.348 (0.234–0.463)
NPV (95% CI)	0.706 (0.489–0.922)	0.527 (0.395–0.659)	0.745 (0.630–0.861)	0.902 (0.838–0.967)	0.429 (0.279–0.578)	0.604 (0.466–0.743)	0.815 (0.711–0.918)	0.965 (0.917–1.013)
Cut-off value	0.565	1.155	1.155	2.095	0.246	0.268	0.301	0.315

*APRI* aspartate aminotransferase to platelet ratio index, *FIB-4* fibrosis index based on four factors, *S0–S4* liver fibrosis stages according to the Scheuer scoring system, *AUC* area under curve, *ACC* accuracy, *SEN* sensitivity, *SPE* specificity, *PPV* positive predictive value, *NPV* negative predictive value

### Comparison of diagnostic values between SIR, T2*L/M, and serum indices (based on adjusted *p*-values using the Holm–Bonferroni method)

Regarding the diagnosis of liver fibrosis S0 vs S1–S4, S0–S1 vs S2–S4, S0–S2 vs S3–S4, Delong’s test showed that the differences in accuracy between the SIR and T2* L/M were not significant, with AUC values of 0.851 vs 0.789 (*p* = 0.803), 0.868 vs 0.828 (*p* = 0.803), and 0.872 vs 0.848 (*p* = 0.803), respectively. However, SIR performed better than T2* L/M in the diagnosis of liver fibrosis S0–S3 vs S4 (*p* = 0.002).

Regarding the diagnosis of liver fibrosis S0 vs S1–S4, Delong’s test showed that the differences in accuracy between the SIR and APRI, SIR, and FIB-4 were not significant, with AUC values of 0.851 vs 0.775 (*p* = 0.183) and 0.851 vs 0.787 (*p* = 0.222), respectively. However, SIR performed better than APRI and FIB-4 in the diagnosis of liver fibrosis S0–S1 vs S2–S4, S0–S2 vs S3–S4, and S0–S3 vs S4 (*p* = 0.011–0.036). Table 4 presents the DeLong’s test results for SIR vs T2 L/M, APRI, and FIB-4 across different liver fibrosis stages.

**Table 4:** DeLong’s test results for SIR vs T2 L/M, APRI, and FIB-4 across different stages of liver fibrosis

Fibrosis stages	T2* L/M			APRI			FIB-4		
	AUC difference^a^	*p*-value	*p*-value^*^	AUC difference	*p*-value	*p*-value^*^	AUC difference	*p*-value	*p*-value^*^
S0 vs S1–S4	−0.062 (−0.171)–(0.048)	0.268	0.803^*^	−0.076 (−0.189)–(0.036)	0.183	0.183^*^	−0.064 (−0.167)–(0.039)	0.222	0.222^*^
S0–S1 vs S2–S4	−0.040 (−0.122)–(0.040)	0.324	0.803^*^	−0.137 (−0.246)–(−0.030)	0.012	0.036^*^	−0.156 (−0.260)–(−0.053)	0.003	0.011^*^
S0–S2 vs S3–S4	−0.024 (−0.105)–(0.056)	0.548	0.803^*^	−0.138 (−0.250)–(−0.026)	0.015	0.036^*^	−0.172 (−0.286)–(−0.060)	0.003	0.011^*^
S0–S3 vs S4	−0.167 (−0.260)–(−0.073)	< 0.001	0.002^*^	−0.162 (−0.280)–(−0.043)	0.007	0.030^*^	−0.177 (−0.303)–(−0.052)	0.006	0.011^*^

*AUC* areas under the curve, *SIR* liver-to-muscle signal intensity ratios, *S0–S4* liver fibrosis stages according to the Scheuer scoring system, T2* L/M liver-to-muscle ratios of T2*, *APRI* aspartate aminotransferase to platelet ratio index, *FIB-4* fibrosis index based on four factors

^a^ AUC differences were calculated using the SIR as the reference

^*^ Adjusted *p*-values using the Holm–Bonferroni method. All subsequent results of DeLong’s test and their interpretations are based on these values

### Correlation analysis

**Fig. 5:**
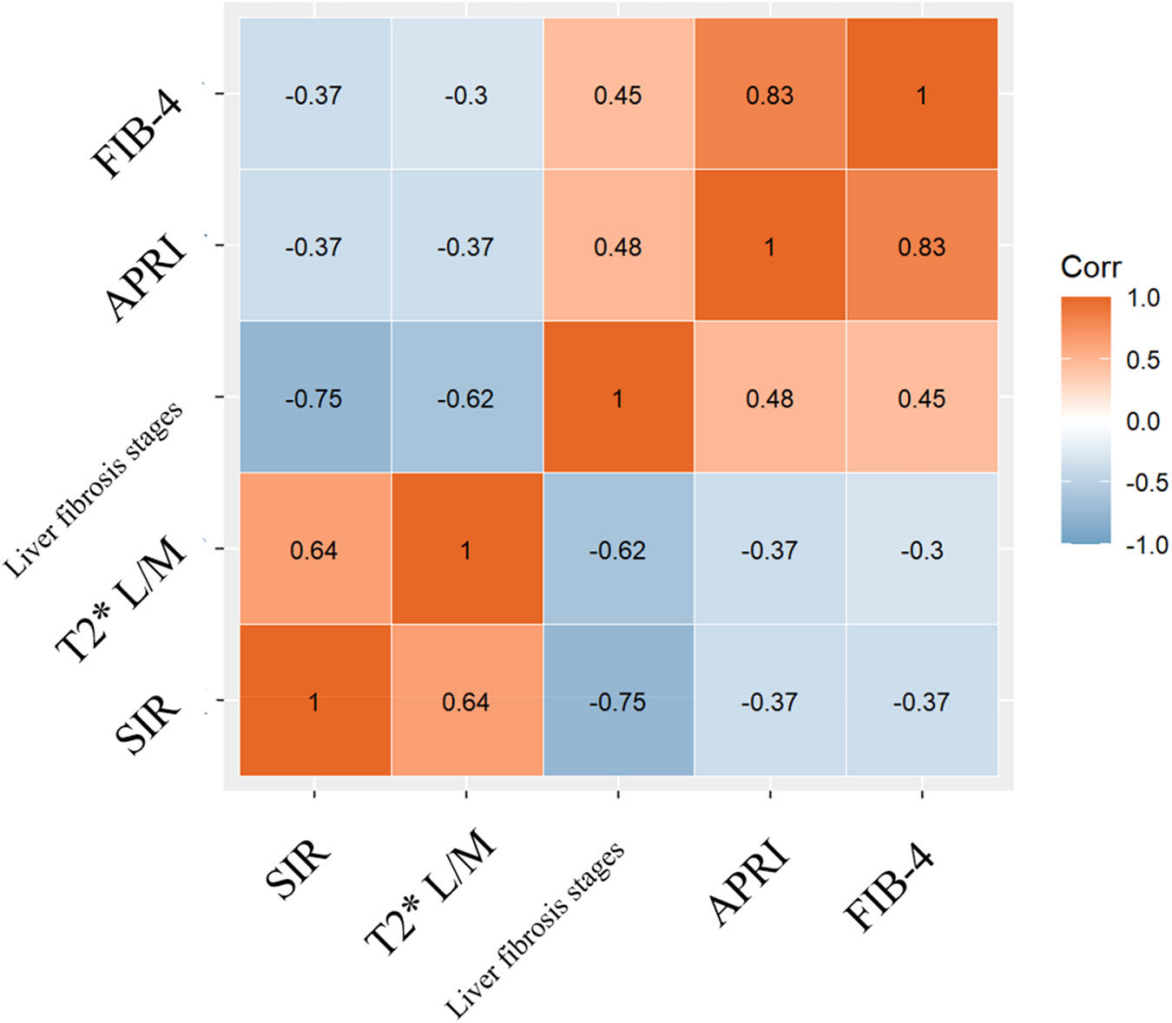
The correlation matrix heat map between SIR, T2* L/M, APRI, FIB-4, and liver fibrosis stages. SIR and T2* L/M showed a negative correlation with the stages of liver fibrosis. Conversely, APRI and FIB-4 exhibited positive correlations with the liver fibrosis stages. SIR, liver-to-muscle signal intensity ratios; APRI, aspartate aminotransferase to platelet ratio index; FIB-4, fibrosis index based on four factors; T2* L/M, liver-to-muscle ratios of T2*

The correlation matrix heat map between SIR, T2*L/M, liver fibrosis stage, APRI, and FIB-4 is illustrated in Fig. 5. A negative correlation was detected between the SIR and various stages of liver fibrosis (*r* = −0.75, *p* < 0.001) as well as between T2*L/M and various stages of liver fibrosis (*r* = −0.62, *p* < 0.001). A positive correlation was observed between the APRI and various stages of liver fibrosis (*r* = 0.48, *p* < 0.001) and between FIB-4 and various stages of liver fibrosis (*r* = 0.45, *p* < 0.001). Additionally, a negative correlation was identified between the SIR and the grade of liver iron deposition (*r* = −0.53, *p* < 0.001).

## Discussion

This study evaluated liver fibrosis staging by quantifying SI on SWI, using histopathological results as the reference standard. Our results showed that SWI-based quantitative liver fibrosis staging using the SIR had good accuracy. In addition, this imaging biomarker outperformed the APRI and FIB-4 in identifying liver fibrosis in S0–S1 vs S2–S4, S0–S2 vs S3–S4, and S0–S3 vs S4. SWI-based SI has proven to be a straightforward and reliable non-invasive assessment method for the accurate diagnosis of liver fibrosis staging. This finding is clinically significant because rapid and accurate quantification of liver fibrosis is crucial for patient prognosis and treatment decisions [6, 25]. The diagnostic utility of SWI likely stems from its high sensitivity to magnetic field changes caused by iron and extracellular collagen deposition [17, 19], which are key pathological features associated with fibrosis progression [18].

The use of SWI for staging liver fibrosis in humans remains in its preliminary stages. Balassy et al [26] initially reported SWI as a viable non-invasive method for detecting liver fibrosis of S0–S1 vs S2–S4 as well as S0–S3 vs S4 in patients with chronic liver disease, with AUC values of 0.920 (95% CI: 0.850–0.970), 0.930 (95% CI: 0.840–0.970) in 80 participants, respectively. The diagnostic performances of S0–S3 vs S4 and S0–S1 vs S2–S4 were comparable, with AUCs of 0.931 and 0.868, respectively. However, their study included a relatively small population and did not measure diagnostic efficacy for all stages of liver fibrosis. Therefore, we further tested the diagnostic performance for liver fibrosis of S0 vs S1–S4 as well as S0–S2 vs S3–S4 based on the SIR in 123 participants, and the results showed comparable diagnostic accuracy, with AUC values of 0.851 (95% CI: 0.759–0.944) and 0.872 (95% CI: 0.802–0.942), respectively.

We further compared the diagnostic effectiveness of the SWI-based SIR with that of serologic indices. SWI is a special diagnostic method with a shorter breath-hold time and faster acquisition, and images can be acquired within only one breath. Its implementation requires only the addition of a single SWI sequence to routine MRI sequences, and the entire process is fully automated. Minimal training for radiologists is also required, focusing solely on the straightforward post-processing steps. More importantly, this method is non-invasive. In this study, ICC analysis and Bland–Altman plots demonstrated strong agreement between different observers in measuring the SIR for diagnosing liver fibrosis staging. Furthermore, this study confirmed that while the difference in diagnostic performance between SIR and serological indicators for stages ≥ S1 was not significant, SIR demonstrated superior diagnostic efficiency for stages ≥ S2, especially in the diagnosis of cirrhosis. Therefore, SWI is a more reliable non-invasive diagnostic method than serological biomarkers for diagnosing various stages of liver fibrosis. The current study generated some unanticipated findings. The cutoff values of S0–S1 vs S2–S4 and S0–S2 vs S3–S4 in FIB-4 had the same results. These results may be explained by some overlap between the serologic indices of adjacent liver fibrosis stages, which partly reflects the limitations of the serologic indices. We also compared the diagnostic effectiveness of the SWI-based SIR with that of T2* L/M. Specifically, MR T2* is widely employed to non-invasively assess and quantify the liver iron content in patients afflicted with hemochromatosis or thalassemia. Prior investigations indicate that T2* exhibits a statistically significant correlation with liver fibrosis stage [17]. While the difference in diagnostic performance was not significant between SIR and T2*L/M for stages S0 vs S1–S4, S0–S1 vs S2–S4, S0–S2 vs S3–S4 in our study, SIR demonstrated superior diagnostic efficiency for stages S0-S3 vs S4. Our results align with the findings reported by Obmann et al [17], in which SWI-based SIR exhibited marginally superior diagnostic performance relative to T2*. These results may be explained by the fact that SWI uses both phase and magnitude information. This involves the multiplication of an original magnitude image with a high-pass filtered-phase mask image, thereby enhancing the contrast and details in the resulting image [15, 16]. While T2* is solely based on the magnitude information, it has the advantage of absolute quantification of the T2*-relaxation times [17]. However, these findings warrant further validation through expanded investigations with larger sample sizes.

SWI is sensitive to iron deposition and has been widely recognized for its clinical value in the central nervous system [27], however, its application in the abdomen is less common. Previous studies [26] using histological results as the standard have confirmed a negative correlation between SIR and liver iron deposition, as well as between SIR and the stages of liver fibrosis (liver fibrosis *r *= −0.81, *p* < 0.001; iron deposition *r* = −0.37, *p* = 0.002). An animal study reported a strong negative correlation between the SIR and the grades of liver iron deposition in 60 rabbits (*r* = −0.81, *p* < 0.001) [28]. Our findings support this pattern, showing that as the stages of liver fibrosis and the grades of iron deposition increased, the SIR gradually decreased (liver fibrosis *r* = −0.75, *p* < 0.001; iron deposition *r* = −0.53, *p* < 0.001). Additionally, Obmanna et al [17] also confirmed the correlation between SWI and liver fibrosis (*r* = −0.47, *p* < 0.001). However, they did not use histopathological evaluation as the best reference standard; instead, they opted for MR elastography. This phenomenon may be explained by the ability of SWI to enhance image contrast by utilizing the susceptibility differences between tissues and combining the phase and magnitude information from MRI signals. Collagen [26] and iron [29] deposition during liver fibrosis can cause decay in the liver SI by accelerating proton dephasing. Increased hepatic iron content alone does not fully encapsulate the pathological transition of fibrosis. Instead, the progressive deposition of collagen is a pivotal driver of fibrotic remodeling. Consequently, SWI-based SIR may serve as a global biomarker of fibrotic burden.

This study has some limitations. First, due to the semiquantitative nature of the Scheuer scoring system, there may be overlaps in histological changes between different stages of liver fibrosis. Therefore, some adjacent liver fibrosis stages did not differ as significantly as expected, which may explain why the SWI-based SIR did not differ significantly in the diagnosis of early liver fibrosis when compared with the serologic indices as well as the results of the one-way analysis of variance. Second, the liver contains trace metallic elements such as copper and zinc, in addition to iron. Although these elements are present in smaller quantities compared to those of iron, their potential impact on the measurement of SIR cannot be entirely ruled out. Furthermore, certain conditions, such as hereditary hemochromatosis, may influence the liver iron deposition and the measurement of SIR. Third, this study did not account for the potential impact of liver steatosis. An ongoing international debate exists about whether hepatic steatosis influences SWI outcomes [17, 26], which could have introduced variability in our findings. Fourth, due to the high prevalence of hepatitis B virus and its complications in China, our cohort predominantly comprised patients with HBV-related hepatopathies and malignancies, rather than NAFLD/non-alcoholic steatohepatitis (NASH) populations, which are more common in Western cohorts. This demographic composition limits the generalizability of our findings to Western or NAFLD/NASH populations. Moreover, the relatively small size of the healthy control cohort and its incomplete demographic matching with the patient population, due to practical constraints, may introduce bias and limit the precision of the comparative analyses. Furthermore, the precise mechanistic contributions of iron deposition and collagen accumulation to variations in SIR values have not been fully elucidated. Notably, histologic correlation for collagen is indirect; future studies should prioritize direct quantification of hepatic collagen to validate its association with SIR and histologic stages of liver fibrosis. Additionally, the lumbar back muscles are affected by iron deposits, steatosis, and muscle atrophy, particularly in patients with chronic liver disease, which can lead to deviations in measurements [30]. We plan to address additional validation in populations with altered muscle composition in future research. Finally, the limited sample size constitutes another constraint of this study. Future studies will include a larger cohort to strengthen the robustness of our results.

In conclusion, our findings demonstrate that the SWI-based liver-to-muscle SIR provides a reproducible and non-invasive method for staging liver fibrosis, with superior diagnostic performance compared with serum biomarkers (APRI and FIB-4), particularly in distinguishing moderate to advanced stages. Given its practicality, we believe this quantitative technique should be considered for integration into routine liver MRI protocols to enhance non-invasive fibrosis assessment, particularly in cases where biopsy is contraindicated or undesired.

## Supplementary information

Supplementary Material

## Data Availability

The data used in this study are not publicly available due to privacy limitations, but can be requested from the corresponding author upon reasonable request.

## Abbreviations

ALT: Alanine aminotransferase
APRI: Aspartate aminotransferase-to-platelet ratio index
AST: Aspartate aminotransferase
AUC: Area under curves
FIB-4: Fibrosis index based on four factors
FOV: Field of view
ICC: Intraclass correlation coefficient
NSA: Number of signals averaged
PLT: Platelet count
ROC: Receiver operating characteristic
ROI: Region of interest
SI: Signal intensity
SIR: Liver-to-muscle signal intensity ratios
SWI: Susceptibility-weighted imaging
T2* L/M: The liver-to-muscle ratios of T2* parametric values
T2WI: T2-weighted images
TE: Echo time
TR: Repetition time
