# Supplementary material for: Assessment of MRI susceptibility-weighted imaging-based liver-to-muscle signal intensity ratios for the staging of liver fibrosis
Source: Insights Imaging. 2026 Jan 28;17:27. doi: 10.1186/s13244-025-02203-2 (PMC12852556; doi:10.1186/s13244-025-02203-2)
Supplement: Supplementary file 1 — Supplementary Material [file 13244_2025_2203_MOESM1_ESM.docx]

**Assessment of MRI susceptibility-weighted imaging-based liver-to-muscle signal intensity ratios for the staging of liver fibrosis**

**(A)** **Supplementary Figures**

**Figure S1** Box-and-whisker plot of SIR and T2* L/M according to Scheuer fibrosis stages

**(B)** **Supplementary Tables**

**Table S1** Distribution of SIR, T2* L/M, APRI, and FIB-4 in various stages of liver fibrosis

**Table S2** One-way analysis of variance results for SIR and T2* L/M





**Figure S1** Box-and-whisker plot^#^ of SIR and T2* L/M according to Scheuer fibrosis stages. SIR and T2* L/M were lower with increasing fibrosis staging. SIR, liver-to-muscle signal intensity ratios of susceptibility-weighted images; T2* L/M, liver-to-muscle ratios of T2*.

^#^ The whiskers in the box-and-whisker plots represent the full data range.

**Table S1** Distribution of SIR, T2* L/M, APRI, and FIB-4 in various stages of liver fibrosis

| **Parameters** | **SIR** | **T2* L/M** | **APRI** | **FIB-4** |  |
| --- | --- | --- | --- | --- | --- |
| S0 | 0.985  (0.960–1.003) | 1.155  (0.990–1.220) | 0.164  (0.130–0.243) | 0.550  (0.405–1.205) | |
| S1 | 0.945  (0.923–0.970) | 1.080  (0.930–1.143) | 0.264  (0.184–0.401) | 1.130  (0.845–2.054) | |
| S2 | 0.920  (0.890–0.930) | 0.950  (0.853–1.045) | 0.296  (0.215–0.455) | 1.370  (0.934–2.268) | |
| S3 | 0.855  (0.830–0.890) | 0.770  (0.605–0.863) | 0.406  (0.262–0.593) | 1.530  (0.929–2.518) | |
| S4 | 0.770  (0.725–0.805) | 0.690  (0.520–0.895) | 0.604  (0.374–1.071) | 2.760  (1.299–3.826) | |
| H value | 69.213 | 49.219 | 29.650 | 27.397 | |
| *P* | <0.001 | <0.001 | <0.001 | <0.001 | |

SIR, liver-to-muscle signal intensity ratios; T2* L/M, liver-to-muscle ratios of T2*; APRI, aspartate aminotransferase to platelet ratio index; FIB-4, fibrosis index based on four factors; S0-S4, liver fibrosis stages according to the Scheuer scoring system. The Kruskal-Wallis H test^#^ showed that there were differences among groups of SIR, as well as T2* L/M, APRI and FIB-4, which were abnormally distributed. Multiple comparisons were adjusted using the Bonferroni method for the Kruskal–Wallis H test.

**Table S2** One-way analysis of variance results for SIR and T2* L/M

|  | **p value of SIR** | **p value of T2* L/M** |
| --- | --- | --- |
| S0 vs. S1 | 0.944 | 0.730 |
| S0 vs. S2 | 0.003 | 0.006 |
| S0 vs. S3 | <0.001 | <0.001 |
| S0 vs. S4 | <0.001 | <0.001 |
| S1 vs. S2 | 0.005 | 0.021 |
| S1 vs. S3 | <0.001 | <0.001 |
| S1 vs. S4 | <0.001 | <0.001 |
| S2 vs. S3 | 0.214 | 0.001 |
| S2 vs. S4 | <0.001 | <0.001 |
| S3 vs. S4 | <0.001 | 0.521 |

SIR, liver-to-muscle signal intensity ratios of susceptibility-weighted images; T2* L/M, liver-to-muscle ratios of T2*; S0-S4, liver fibrosis stages according to the Scheuer scoring system.
